# Video Demonstration of Conversion from One Anastomosis Gastric Bypass to Sleeve Gastrectomy in a Patient with Protein Energy Malnutrition

**DOI:** 10.1007/s11695-024-07456-2

**Published:** 2024-08-24

**Authors:** Mohamed Hany, Mohamed Ibrahim, Anwar Ashraf Abouelnasr, Bart Torensma

**Affiliations:** 1 Department of Surgery, Medical Research Institute, Alexandria University, 165 Horreya Avenue, Hadara, 21561 Alexandria Egypt; 2 Madina Women’s Hospital, Alexandria University, Alexandria, Egypt; 3 Leiden University Medical Center (LUMC), Leiden, The Netherlands

**Keywords:** Conversion surgery, One anastomosis gastric bypass, Sleeve gastrectomy diarrhea, Protein energy malnutrition

## Introduction

One-anastomosis gastric bypass (OAGB) is recognized as a metabolic bariatric surgery (MBS) by the International Federation for the Surgery of Obesity and Metabolic Disorders (IFSO) [1] and the American Society for Metabolic and Bariatric Surgery (ASMBS) [2]. It is noted for high weight loss (WL) rates and resolving medical issues [3]. Studies indicate that OAGB provides superior WL results, fewer complications, and quicker procedures than Roux en Y gastric bypass (RYGB) [4].

### OAGB and Protein-energy Malnutrition

Despite its benefits, OAGB can cause long-term issues like chronic bile reflux, malnutrition, dumping syndrome, hypoglycemia, and marginal ulcers. Notably, severe protein-energy malnutrition (PEM), with an average incidence of 26%, but also described in a systematic review (SR) from 28.6 to 54.8% after OAGB, is a severe complication that may require surgery [5]. Nevertheless, the literature does not consistently define PEM, its severity, or criteria for revision surgery. The etiology of PEM is likely multifactorial, involving altered gut-hormonal dynamics and other factors such as new-onset anorexia, psychiatric disorders, non-adherence to nutritional guidelines, and secondary pathologies like colitis, celiac disease, or small intestinal bacterial overgrowth [6].

Furthermore, there is no consensus on the appropriate biliopancreatic limb (BPL) length in OAGB. In two systematic reviews comprising 318 and 1075 patients, respectively, the outcomes of OAGB were evaluated in patients with a BMI ≥ 50 kg/m^2^ and those undergoing OAGB as revisional MBS. These studies reported no significant cases of malnutrition despite variations in BPL lengths ranging from 150 to 250 cm [7, 8]. A retrospective study involving 925 OAGB patients found that 2.3% required revisional surgery following the initial procedure. Of these, 0.5% necessitated intervention due to severe diarrhea, which was managed by shortening the BPL to 150 cm [9]. Therefore, most authors endorsed a fixed length of 200 cm [10, 11]. Some authors proposed a shorter BPL (150 cm) to avoid profound nutritional defects and increased causes of diarrhea [12].

### Reversal to the Original Anatomy

Reversal to the original anatomy can result in weight regain and has technical risks like leaks and stenosis. The choice of revisional surgery after OAGB depends on specific needs. Conversion to RYGB is typical for severe bile reflux or persistent marginal ulcers but carries risks of malabsorption in severely malnourished patients.

LSG is preferred for severe protein-calorie malnutrition. It aims to maintain weight loss. It effectively resolves nutritional deficiencies while preserving body mass index [13, 14]. While the conversion of OAGB to LSG has been described in the literature, we aimed to present a video of the case.

### Case Presentation

A 54-year-old female with a history of OAGB was referred to our clinic, Madina Women’s Hospital, Alexandria, Egypt, and presented with weakness, dizziness, and frequent watery diarrhea three months after her operation in June 2020. Initially, her body mass index (BMI) was 41 kg/m^2^, and her BMI decreased to 26 kg/m^2^ post-surgery despite treatments with loperamide and pancreatic enzymes. The patient did not consistently take her multivitamins. Proton pump inhibitors (PPIs) were taken regularly for the first 6 months post-surgery and then used occasionally to manage gastric complaints such as heartburn and pain. Furthermore, she was thoroughly evaluated by a multidisciplinary team (MDT) before surgery. There were no other medical-associated problems, and the patient did not smoke. Additionally, her mental health status was stable, with no issues related to alcohol use or other associated medical problems. Multiple imaging studies, including upper and lower GI (GI) endoscopy, computed tomography (CT), and abdominal ultrasound, ruled out common GI issues. The gastric pouch volume was measured to be 250 cc, as indicated by the CT scan, which showed dilation. The pouch appeared short, approximately 10 cm. However, during the preoperative endoscopy, we did not observe excessive bile in the gastric pouch or the esophagus. Lab tests showed several deficiencies: albumin at 3 gm/dL, ferritin at 9 ng/mL, hemoglobin at 9.9 gm/dL, calcium at 8.4 mg/dL, and vitamin D3 at 14 nmol/L. Treatment included nutritional support to correct these imbalances, successfully increasing her albumin to 3.8 g/dL, hemoglobin to 10.7 g/dL, and calcium to 8.7 mg/dL within a month. The patient underwent a glucose breath test, which yielded negative results. Thus, no treatment for small intestinal bacterial overgrowth (SIBO) was required. Furthermore, aminoleban and pravotin were administered to address hypoalbuminemia and iron deficiency anemia. The patient was offered several surgical options, including reversal to normal anatomy, shortening the biliopancreatic limb, and conversion to RYGB. She opted to reverse the surgery to maintain her weight. The patient provided written informed consent after extensive discussions about potential complications and intraoperative adjustments.

### Reversal Surgery

In January 2022, the patient underwent a laparoscopic reversal of OAGB. The surgery started with adhesiolysis and measuring the entire small intestine, revealing a BPL of 250 cm and a common channel of 320 cm. The reversal involved horizontally dividing the gastrojejunostomy (GJ) anastomosis and constructing a side-to-side jejunojejunostomy to avoid stenosis. The remnant GJ was removed, and the proximal gastric pouch was resized using a 40-Fr tube. A side-to-side gastro gastrostomy (GG) was established between the gastric pouch and the antrum, followed by creating a new sleeve with linear staplers and closing the GG stoma with a 3/0 PDS V-Loc barbed suture (Covidien, Mansfield, MA, USA). Staple lines were reinforced and tested for leaks. The operation took 100 min.

### Postoperative Course and Follow-up

The postoperative period was uneventful, with the patient tolerating oral fluids and maintaining stable vital signs. The tube drain was removed on the fourth day, and a CT scan confirmed no contrast leakage. At 1 month, she was consuming fluids and a soft diet without previous symptoms of diarrhea. Significant lab improvements at 1 year included hemoglobin at 11.2 g/dL, calcium at 9.2 mg/dL, and albumin at 4 g/dL. Her BMI stabilized at 27 kg/m^2^, reflecting a successful surgical outcome and management.

## Conclusion

Conversion from an OAGB to LSG is a viable option for managing protein-energy malnutrition, provided proper preparation is made. This approach maintains weight loss while addressing nutritional issues. Tailoring the management plan to each patient’s specific needs ensures optimal outcomes.

## Supplementary Information

Below is the link to the electronic supplementary material.

Supplementary file1 (MP4 268017 KB)

## Data Availability

Data is available with the corresponding author.
